# The importance of childhood social capitals in the future well-being of children

**DOI:** 10.3389/fpsyg.2024.1389269

**Published:** 2024-07-25

**Authors:** Chihiro Hosoda, Zhang YunFeng, Junji Watanabe, Kazushi Maruya, Rokuro Tabuchi, Kenchi Hosokawa, Takuto Matsuhashi

**Affiliations:** ^1^Graduate School of Information Sciences, Tohoku University, Sendai, Miyagi, Japan; ^2^Institute of Development, Aging and Cancer, Tohoku University, Sendai, Miyagi, Japan; ^3^NTT Communication Science Laboratories, Atsugi, Kanagawa, Japan; ^4^Faculty of Human Sciences, Sophia University, Chiyoda, Tokyo, Japan

**Keywords:** parenting, social capital, well-being, child–parent relationship, non-parental adults

## Abstract

**Introduction:**

Elucidating the enduring effects of childhood social capital is vital for shaping future well-being. Here, we investigate the impact of childhood social capital (SC) and parental engagement on adult psychological well-being and cognitive performance.

**Methods:**

Utilizing a cohort of 292 Japanese university students, we assessed the influences of SC and parental time during childhood on adult outcomes. Participants retrospectively reported their early childhood experiences, focusing on social interactions and parental involvement.

**Results:**

Our findings reveal a significant positive correlation between childhood SC and adult subjective well-being, particularly in its positive dimensions. Additionally, maternal involvement during childhood is associated with cognitive abilities in adulthood, as measured by Raven’s Advanced Progressive Matrices. Hierarchical multiple linear regression analysis highlights the substantial impact of childhood SC on adult well-being, elucidating the intricate interplay between social and parental contributions to developmental trajectories.

**Discussion:**

These results highlight the crucial roles of both parental and non-parental figures in fostering social, emotional, and cognitive development from childhood to adulthood, underscoring the importance of nurturing supportive relationships throughout early life to promote positive developmental outcomes.

## Introduction

Parents remain one of the most important factors influencing children’s well-being (Rees and Dinisman, 2015; Fallesen and Gähler, 2020). Positive parent–child relationships and the length of quality time spent with parents are associated with positive social and emotional outcomes in children and directly affect children’s ability to manage their worlds socially, emotionally, and intellectually (Frosch et al., 2021). They also impact their social, emotional, and cognitive functioning, school achievement, and mental and physical health (Li and Guo, 2023).

Recent research also highlights the pivotal role of non-parental figures, such as neighbors, peers, and friends, in shaping children’s well-being (Minh et al., 2017). Aspillaga et al. (2022) demonstrated that children’s sense of belonging, closeness, and support within peer groups and from adults in educational and neighborhood settings significantly contribute to their overall well-being. Furthermore, studies emphasize the critical role of crucial non-parental adults in supporting foster youth during their transition to adulthood by providing emotional and financial support, thereby mitigating negative outcomes; (Haddad et al., 2011; Duke et al., 2017). These findings suggest that non-parental relationships substantially impact individual future development and well-being.

Moreover, research by Coulton and Spilsbury (2014) indicates that adverse childhood environments (ACEs), characterized by social and economic isolation, correlate with a heightened risk of negative outcomes, including academic failure, health issues, and social challenges. Research has shown that positive interventions and stable, nurturing relationships outside the family unit can significantly mitigate the long-term negative effects associated with ACEs (Bethell et al., 2014; Crouch et al., 2019). These interventions provide essential emotional and social support, helping to develop resilience and coping strategies in children. Daycare centers and supportive educational programs play a critical role in offering a safe and structured environment where children can learn, socialize, and grow in a positive direction, away from the stressors that may dominate their home environments (Sylva et al., 2004). Furthermore, engagement in high-quality early childhood education has been linked to improved academic and social outcomes, particularly for children from disadvantaged backgrounds (Cunha and Heckman, 2010) Thus, non-parental care and support act as vital protective factors that can counteract the adverse effects of challenging early life experiences, underscoring the importance of accessible, high-quality childcare and support services for vulnerable children. Conversely, perceptions of positive early caregiving contribute to future relationships and are positively associated with fewer depressive symptoms over time in the adult life span (Chopik and Edelstein, 2019). These perspectives collectively underscore the value of non-parental connections in providing children as non-parental social capital (NPSC) may also be present in the child’s well-being and have a long-term impact on people’s development. By offering access to diverse resources, role models, emotional support, and opportunities, these connections play a critical role in shaping children’s trajectories and outcomes.

However, these studies are predominantly cross-sectional and often focused on specific demographics like foster youth or unusual childhood, limiting their generalizability to understanding the long-term effects of NPSC on normal adolescent well-being.

Social Capital (SC) encompasses the breadth and depth of interpersonal connections alongside the collective attitudes and behaviors that foster a cohesive and well-functioning society (Tuominen and Haanpää, 2022). Putnam’s (2000) three-dimensional definition of SC, encompassing social relations, trust in others, and norms of reciprocity, is recognized among the foremost theories on the subject. The burgeoning literature links SC to diverse health outcomes and well-being measures (Helliwell and Putnam, 2004; Kawachi et al., 2004; Huppert and So, 2013; Haanpää et al., 2019), though the evidence strength varies based on SC conceptualization and measurement, study population demographics, analysis levels, and countries’ income levels.

The established correlation between social capital and enhanced life quality, health, and life satisfaction is well-documented (Hommerich and Tiefenbach, 2018; Xu et al., 2022). However, there remains a significant gap in our understanding of the long-term effects of childhood SC on adult well-being. Prevailing studies predominantly categorize childhood experiences into binary classifications of positive or negative, thus offering a limited perspective on their long-term influence on adult well-being. Although Han et al. (2023) found a modest link between Positive Childhood Experiences (PCEs) and reduced adversity in adulthood, their study did not differentiate between positive or negative experiences stemming from parental interactions versus those with non-parental figures. Furthermore, they did not thoroughly investigate the distinct effects of social capital on adult well-being. Oshio et al. (2013) investigated the influence of childhood interpersonal adversity on adult subjective well-being (SWB), with a particular focus on the roles of social support and socioeconomic status (SES) as mediating and moderating factors. They found that negative impact, social support, and SES mediated childhood adversity’s effect on adulthood SWB. Tuominen and Haanpää (2022) explored young people’s social capital at 12–13 years, highlighting the significant influence of trust, reciprocity, and social networks, particularly within families and communities, on adolescents’ well-being. These studies contribute to understanding the variances in well-being but do not fully address the role of SC in childhood.

This study endeavors to bridge the existing research gap by concentrating on the role of SC during childhood and its enduring effects on well-being in adulthood. We intricately examine how SC, in conjunction with parental influences, molds the developmental paths of individuals from childhood to adulthood. Our approach broadens the scope of understanding childhood experiences, moving beyond the traditional dichotomy of positive and negative experiences to a more comprehensive view of childhood social capital and its lasting impact. Additionally, in light of the established significance of parental involvement in shaping individual cognitive abilities such as problem-solving and intelligence; (Klocke and Stadtmüller, 2019; Zhang et al., 2020), we also conduct further analysis to explore whether SC, the time spent with their mother or father in childhood is associated with the cognitive skills in the adulthood.

## Materials and methods

### Participants

Through a web survey, a total of 292 Japanese university students were surveyed, with an average age of 19.5 years (standard deviation = 19.5 ± 1.2, age range 18–21 years). The survey was conducted online and announced via the communication channels of Tohoku University. Participants responded to questionnaire items including measures of subjective well-being, social capital during childhood, intelligence quotient, and assessments of time spent with parents.

No participant reported having a history of psychiatric or neurological conditions. Ethical approval was obtained from the Research Ethics Board of Tohoku University.

### Measures

#### Social capital in childhood

The assessment of SC consists of four questions, two each on trust and reciprocity (Kawachi et al., 1997; Kawachi, 1999). In the Kawachi study, in all questions, the term “people” referred to the entire relationship including family around the participants, but in our study, we replaced the term “people (adults) outside the family,” in all questions, to emphasize the SC outside the family. Each item was answered using a 4-point scale as follows: 4 = ‘I strongly agree,’ 3 = ‘I somewhat agree’, 2 = ‘I somewhat disagree’ and 1 = ‘I strongly disagree.’ A higher total score indicates a higher SC. Participants were asked to recall their childhood (early elementary school years) and answered the questions. The early elementary school years were selected as the focal point for assessing SC in childhood due to significant developmental milestones occurring during this stage. This period is critical as children begin transitioning from a predominantly home-centered life to school, where they encounter new social challenges and opportunities (Züchner, 2012). During these years, children’s social and cognitive abilities undergo rapid development, enabling them to form and navigate complex social networks. According to Ladd (1999), the relationships and social skills established during this phase are foundational, strongly influencing later social competencies and network benefits. Earlier assessments, prior to school entry, might not accurately reflect children’s ability to form enduring social networks due to less developed social cognition (Thompson, 2006).

#### Subjective well-being

SWB can be quantified using the Japanese version of the WHO’s Subjective Wellbeing Inventory (SUBI) (Sell, 1992) comprised of 15 items (*α* = 0.93). SUBI is a self-report questionnaire used to assess the degree of an individual’s physical, mental and social well-being and enables the measurement of two types of SWB independently: positive affects (PA) and negative affects (NA). 15 items are divided into 5 subscales: Confidence, Transcendent, Positive Affect, Expectation-Achievement Contingency (EAC), and Negative Affect. PA comprises 12 items included in subscales Confidence, Transcendent, Positive Affect, EAC, and NA comprises 3 items included in subscales Negative Affect. Each item was answered using a 4-point scale as follows: 4 = ‘I strongly agree,’ 3 = ‘I somewhat agree’, 2 = ‘I somewhat disagree’ and 1 = ‘I strongly disagree.’

#### Spent time with mother or father

Given the theoretical importance of parental accessibility for children’s developmental outcomes, our study sought to measure the average amount of time per day that parents are accessible to their children. Accessibility, in this context, refers to both the physical presence of the parent and their availability to provide emotional support and supervision throughout the day. The significance of parental accessibility is well-documented in the literature. Social control theory posits that parental supervision, facilitated by parental accessibility, is crucial for protecting children from risk-taking behaviors (Barnes and Farrell, 1992; Longmore et al., 2009) and promoting academic achievement (Amato and Fowler, 2002). Additionally, cultural norms emphasize that “being there” is a key marker of good parenting, providing children with a sense of security and immediate emotional support (Garey, 1996; Hays, 1996; Kurz, 2000; Snyder, 2007). To quantify this aspect of parenting, we included a survey question asking participants to report the average amount of time per day that they are physically present and accessible to their children. This measure was designed to capture both the quantity of time and the availability of parents for immediate interaction and support. In order to measure the amount of time that both fathers and mothers spend with their children, participants were asked to report the amount of time that mothers and fathers spent at home with them, on average, each day over the course of their own waking hours in childhood (early elementary school years) The response of four choices were as follows: 1 = ‘Most of the time at home, 2 = 8 h or more’, 3 = ‘More than 6 h but less than 8 h’, 4 = ‘More than 4 h but less than 6 h’,5 = ‘More than 2 h but less than 4 h’ and 6 = ‘less than 2 h.

#### Raven’s Advanced Progressive Matrix

The Raven’s Advanced Progressive Matrix (RAPM; Raven, 2000), a widely used measure of non-verbal intelligence (Melby-Lervåg and Hulme, 2013), comprises 25 items, featuring a 3 × 3 set of figural patterns that lacks the bottom-right pattern, along with eight response options that may conceivably correspond to an absent pattern (Raven, 1981). Participants must find the rule underlying the pattern distribution and select the correct answer from the choices.

### Data analysis

We first aimed to examine the association between the SWB-PA and SWB-NA scores obtained from the SUBI scale, respectively, and SC in childhood obtained from the modified version of the SC scale (Kawachi et al., 1997; Kawachi, 1999), time spent with the mother, time spent with the father using 6-point scales. (For details of the scales, see the respective descriptions in the Measure section.) For this purpose, Spearman’s rank correlation coefficient with Bonferroni correction was employed. This non-parametric measure was chosen due to the non-normal distribution of the data, and it assesses the relationship between SC in childhood, time spent with the mother, time spent with the father, and SWB-PA or SWB-NA in adulthood. Secondly, the hierarchical multiple linear regression analyses to investigate the independent contributions of SC score, and time spent with mother or father in childhood to SWB-positive in adulthood or SWB-negative in adulthood was conducted. Additionally, the participants were divided into two groups by median SC score in childhood (early elementary school years) and compared SWB at the time of the answer (adulthood) in the high and low SC groups. Also, to examine the relationship between time spent with mother or father in childhood (early elementary school years) and SWB in adulthood, we similarly divided participants into two groups based on the median score of time spent with mother or father in childhood and compared SWB at the time of response (adulthood) between the long and short groups.

Also, we aimed to examine the association between the fluid intelligence scores obtained from the Raven’s Advanced Progressive Matrix, and SC in childhood, time spent with the mother, and time spent with the father using Spearman’s rank correlation coefficient with Bonferroni correction. Next, the participants were divided into two groups by median SC score in childhood and compared the score of the Raven’s Advanced Progressive Matrix at the time of the answer (adulthood) in the high and low SC groups. Also, to examine the relationship between time spent with mother or father in childhood and the score of the Raven’s Advanced Progressive Matrix in adulthood, we similarly divided participants into two groups based on the median score of time spent with mother or father in childhood and compared the score of the Raven’s Advanced Progressive Matrix at the time of response (adulthood) between the long and short groups. Descriptive statistics, including means and standard deviations, were calculated, and normality was assessed using the Kolmogorov–Smirnov test. Group differences were analyzed using t-tests with multiple comparisons. The significance level was set at *p* < 0.05. Statistical analyses were performed using IBM SPSS version 25.

## Results

### Associations between social capital in childhood and well-being

Mean SC, mean time spent with mother, time spent with father, subjective well-being positive affect (SWB-PA) scores, and subjective well-being negative effect (SWB-NA) scores were 11.3 ± 1.5, 1.3 ± 1.4, 3.6 ± 1.0, 40.5 ± 5.3, and 6.9 ± 2.1, respectively, (Table 1).

**Table 1:** Summary of characteristics and descriptive statistics.

	Mean	SD	Min.	Max
Age	19.5	1.2	18	25
Sex (male: female)	211: 81			
Social capital	11.3	1.5	7	16
*Spent time*				
Mother	1.3	1.4	0	5
Father	3.6	1.0	0	5
*SWBI score*				
Positive	40.5	5.3	24	53
Negative	6.9	2.1	3	12

Spent time 1 = ‘Most of the time at home’, 2 = ‘8 h or more’, 3 = ‘More than 6 h but less than 8 h’, 4 = ‘More than 4 h but less than 6 h’, 5 = ‘More than 2 h but less than 4 h’ and 6 = ‘less than 2 h’.

The Spearman’s rank correlation coefficient revealed a significant positive correlation between the SC in childhood and the SWB-PA (*r* = 0.16, *p* = 0.034) (Table 2). No significant correlation was detected between the additional variables, specifically the time spent with the mother and the time spent with the father, and SWB-PA. Additionally, no variables exhibited significant correlations with SWB-NA (Table 2).

**Table 2:** Spearman’s rank correlation with Bonferroni correction in subjective well-being and social capital in childhood, spent time with mother, and spent time with father.

	SWB positive		SWB negative	
	*r*	*p*-value	*r*	*p*-value
Social capital in childhood	0.162	0.034	−0.120	n.s.
Spent time with mother	0.004	n.s	0.079	n.s.
Spent time with father	−0.136	n.s.	0.140	n.s.

**Figure 1:**
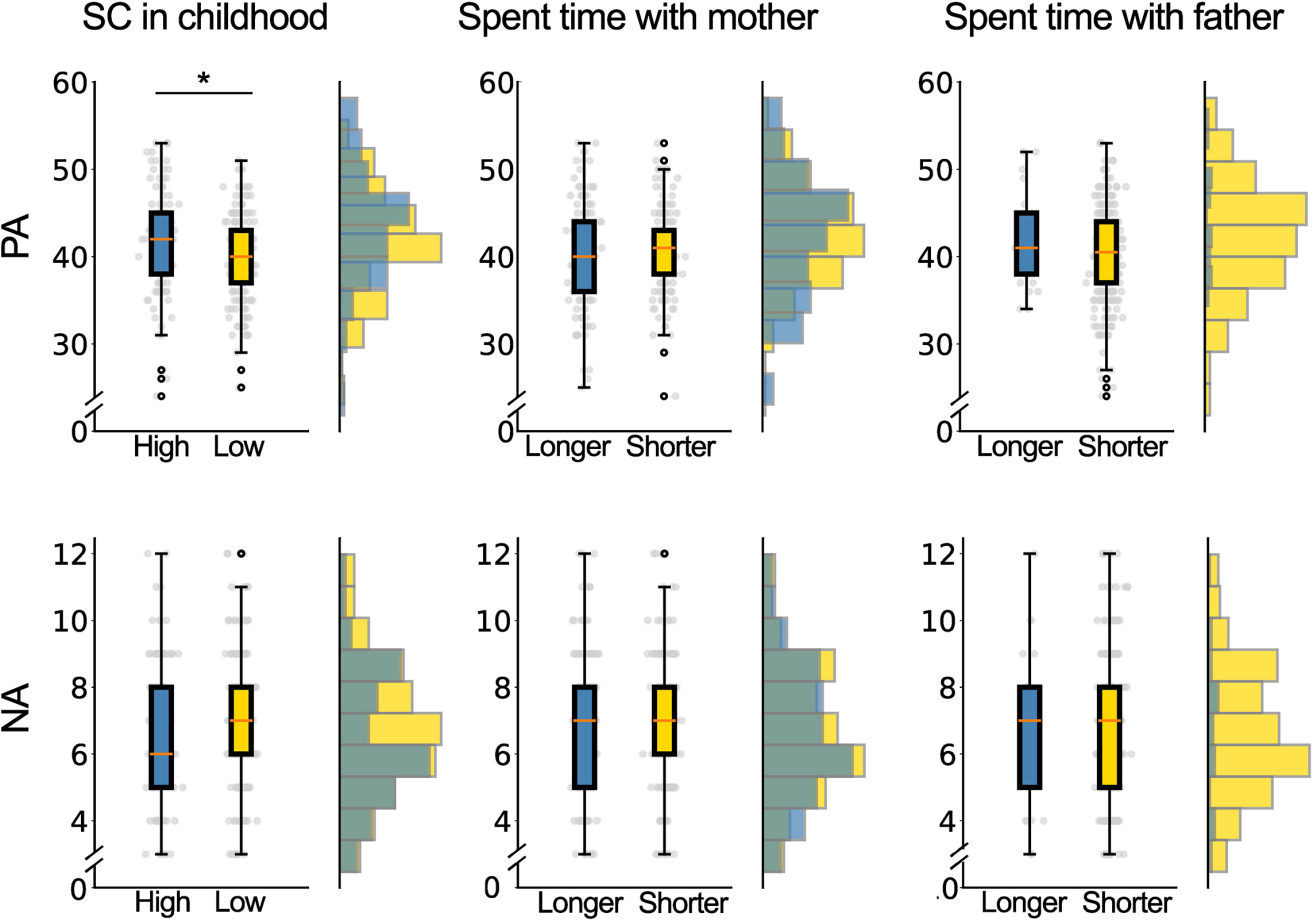
Comparison of the subjective well-being scores in adulthood for each difference in social capital in childhood, spent time with mother and spent time with father in childhood. The left panel shows a comparison of Subjective Well-Being (SWB) scores between high (*N* = 119) and low (*N* = 173) social capital (SC) in childhood. The vertical axis represents the comprehensive SWB subscale score, where the blue plot indicates the high-SC group and the yellow plot indicates the low-SC group. A significant difference was shown in the positive affects (PA) between the high-SC and low-SC. The boxplot displays the SWB subscale scores for PA and NA (negative affects) in the high-SC and the low-SC. The middle panel compares SWB scores between the longer parental time group (*N* = 142) and the shorter parental time group (*N* = 150) for time spent with mothers, with the blue plot representing the longer group and the yellow plot representing the shorter group. The right panel shows the comparison of SWB scores between the longer parental time group (*N* = 25) and the shorter parental time group (*N* = 267) for time spent time with fathers. No group differences were found in the SWB score in the difference in the paternal time (*p*s > 0.05).

To analyze the independent contributions of SC score, time spent with mother, and time spent with father in childhood to SWB-PA or SWB-NA in adulthood, we performed hierarchical multiple linear regression analyses using the stepwise method. SC was significantly related to SWB-PA or SWB-NA (*β* = 0.20, *p* < 0.001; *β* = 0.17, *p* = 0.025). The models explained 26.0% (*R*^2^), *F* (1,291) = 11.95, *p* < 0.001 for SWB-positive and 2% (*R*^2^), *F* (1,291) = 4.04, *p* < 0.028 for SWB-negative. Additionally, participants were divided into two groups based on the median SC score. Participants were stratified into two groups based on the median SC score in childhood. SWB-PA scores in adulthood were 41.5 ± 5.6 (*N* = 119) for high SC and 39.8 ± 4.9 (*N* = 173) for low SC (Figure 1, upper-left panel, Table 2). T-tests revealed a significantly higher SWB-PA score in the high SC group compared to the low SC group (*p* = 0.006). SWB-NA scores in adulthood were 6.7 ± 2.1 for high SC and 7.1 ± 2.1 for low SC, with no significant difference observed (*p* = 0.108) (Figure 1, lower-left panel, Table 2).

Similarly, Spearman’s rank correlation coefficient revealed a significant positive correlation between the time spent with the mother in childhood and the Ravens’ score (*r* = −0.21, *p* = 0.001). No significant correlation was detected between the additional variables, specifically the time spent with the father and the time spent with the father (*r* = 0.00, *p* = 0.92), and SC (*r* = 0.01, *p* = 0.75). Additionally, no variables exhibited significant correlations with SWB-NA participants who were categorized based on the median time spent with their mothers during childhood. SWB-PA scores in adulthood were 40.3 ± 5.8 (*N* = 142) for a long time spent with the mother and 40.7 ± 4.7 (*N* = 150) for a short time spent with the mother, with no significant group difference (*p* = 0.51) (Figure 1, upper-middle panel). SWB-NA scores in adulthood were 6.9 ± 2.1 for a long time spent with the mother and 6.9 ± 2.0 for a short time spent with the mother, showing no significant group difference (*p* = 0.89) (Figure 1, lower-right panel).

Lastly, participants were divided based on the median time spent with their fathers during childhood. The Kolmogorov–Smirnov test indicated that the data exhibited a normal distribution. SWB-PA scores in adulthood were 41.9 ± 5.2 (*N* = 25) for a long time spent with the father and 41.9 ± 5.2 (*N* = 267) for a short time spent with the father, with no significant group difference (*p* = 0.16) (Figure 1, upper-right panel). SWB-NA scores in adulthood were 6.8 ± 2.4 for a long time spent with the father and 6.9 ± 2.1 for a short time spent with the father, demonstrating no significant group difference (*p* = 0.83) (Figure 1, lower-right panel) (Table 3).

**Table 3:** Means and SDs of subject demographics and subitems in subjective well-being, and statistics in each group.

Subjective well-being	Group		Statistics
*Social capital in childhood*			
	High (*N* = 119)	Low (*N* = 173)	*p*-value
Positive	41.5 ± 5.6	39.8 ± 4.9	0.006
Negative	6.7 ± 2.1	7.1 ± 2.1	0.108
*Spent time with mother*			
	Long (*N* = 142)	Short (*N* = 150)	*p*-value
Positive	40.3 ± 5.8	40.7 ± 4.7	0.513
Negative	6.9 ± 2.1	6.9 ± 2.0	0.846
*Spent time with father*			
	Long (*N* = 25)	Short (*N* = 267)	*p*-value
Positive	41.9 ± 5.2	40.4 ± 5.3	0.160
Negative	6.8 ± 2.4	6.9 ± 2.1	0.820

### The relationship between parental time and cognitive ability

To explore how parenting involvement affects cognitive ability which is a non-verbal fluid intelligence test, we did the Spearman’s rank correlation coefficient and revealed a significant positive correlation between the time with mother in childhood and the Score of Ravens Advanced Matrix (*r* = 0.19, *p* = 0.001). Additionally, we compared the correctness of RPM tasks between the high-SC group and the low-SC group, as well as between the long and short-parental time groups. The Wilcoxon test revealed no significant difference between SC and the amount of time spent with the father (*p*s > 0.42). However, the participants who spent more time with their mothers in childhood showed significantly higher cognitive ability than those who spent less time with their mothers. (*p* = 0.003).

## Discussion

**Figure 2:**
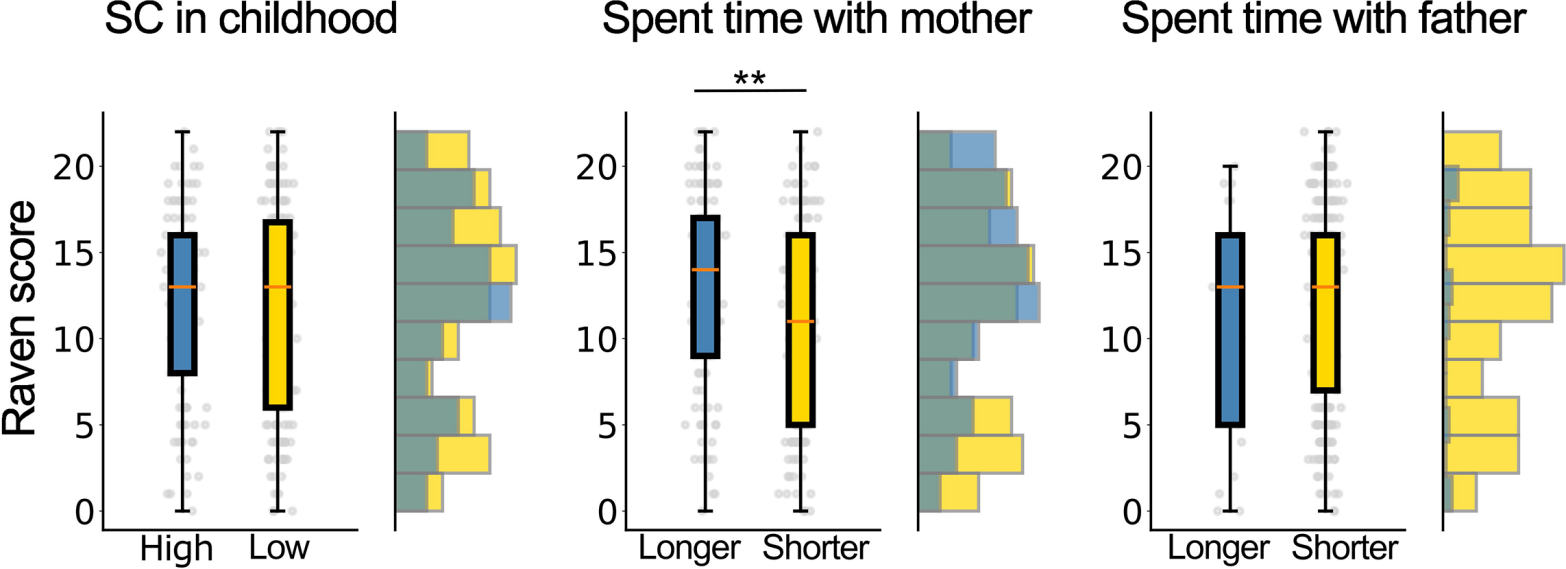
Comparison of Raven’s Progressive Matrices performance based on differences in social capital, time spent with mother, and time spent with father in childhood. The left bar plot shows the number of correct answers on the Raven’s Progressive Matrices (RPM) task for the high-SC (blue) and low-SC (yellow), the middle plot compares the longer (blue) and shorter (yellow) groups for time spent with mothers, and the right bar plot the longer (blue) and shorter (yellow) groups for time spent with father. Only the middle bar showed a significant difference after correction for multiple testing (*p* < 0.05 / 3). **indicates significant differences (*p* < 0.01).

Our findings highlight the significant role of non-parental social capital in enhancing children’s well-being and cognitive development. We demonstrated that diverse social interactions, beyond those with parents, contribute positively to children’s social and emotional growth. This underscores the importance of broad social networks that include both familial and non-familial adults in supporting the developmental trajectories of children. Additionally, our results suggest that the amount of time parents spend with their children may also influence academic performance, further suggesting the complex interplay between various forms of social capital and child development (Figure 2).

The association between non-parental involvement and enhanced well-being suggests that the breadth and quality of social interactions children experience with caregivers, educators, and peers or adults of their community outside their home environment contribute to a child’s sense of belonging, self-esteem, and emotional support (Putnam, 2000) These relationships provide diverse experiences and coping mechanisms, enriching a child’s social and emotional development, which are crucial components of overall wellbeing (Coleman, 1988). This concept aligns with the idea of social capital as a critical resource, where networks of relationships provide children with various forms of support, knowledge, and opportunities for socialization and learning. Herbst provides evidence that non-parental childcare settings can offer enriching experiences that contribute positively to child development, highlighting the role of these settings in compensating for the “summer participation dip” in learning and engagement (Herbst, 2013). This finding suggests that non-parental entities’ structured and socializing environments can offer developmental benefits not always replicated within parental care. Similarly, the work of Elmore et al. examining the interplay of ACEs and resiliency on depression outcomes further amplifies the complexity of factors influencing child and youth well-being beyond the immediate family sphere (Elmore et al., 2020). Previous research also supported the theoretical framework proposed by Harris who argued that the child’s environment extends beyond the family and includes significant influences from peer groups and societal structures (Harris, 1995). Moreover, Conger and Donnellan emphasize the importance of broader social environments in shaping individual development trajectories (Conger and Donnellan, 2007).

Recent studies have emphasized the profound impact of childhood experiences on future mental health and well-being. PCEs, including supportive family interactions and caring relationships with friends and community members, play a critical role in shaping adult mental health and relational well-being (Bethell et al., 2019; Huang et al., 2023). Many studies found that the prevalence of ACEs not only strongly influences poor academic performance, poor health outcomes, and certain diseases but also influences future health and well-being (Hughes et al., 2016; Trivedi et al., 2021; Webster, 2022). Daines et al. (2021) point out that both PCEs and ACEs affect individual physical and mental health in adulthood, although the effects on family health are less explored. Research by the Bethell et al. (2019) demonstrates that PCEs are associated with reduced risks of adult depression and poor mental health and increase the likelihood of having healthy adult relationships. Furthermore, the presence of trusted non-parental adults during childhood is recognized for enhancing resilience and social skills development, with significant long-term implications (Ashton et al., 2021). These previous studies showed the impact of children and their overall mixed relationships, both family and non-family relationships, on well-being. The present study showed that trusting networks with others. May also be important for the future well-being of children this may be due to the fact that children needed support from not only parents but non-parental adults and they had an important influence different from that of the parents on the emotional and psychological health of their children while having a long impact.

Our findings are consistent with the studies on the importance of diverse social environments for child development. The systematic protocol by Johnstone et al. (2020), on nature-based early childhood education underscores the potential of varied educational settings to support child health, well-being, and development, reinforcing the idea that non-parental environments contribute uniquely to child well-being. Furthermore, the coupling of well-being and cognition during development provides an empirical basis for understanding how non-parental involvement might influence well-being through cognitive and social mechanisms, suggesting a multifaceted relationship between environmental influences and developmental outcomes (Fuhrmann et al., 2022).

Conversely, the association of time spent with a mother with higher nonverbal IQ, as measured by tasks Raven’s matrices, suggests that direct parental involvement plays a critical role in cognitive development. Studies consistently demonstrate that positive maternal behaviors, such as verbal engagement, emotional support (Brooks-Gunn and Markman, 2005), and cognitive stimulation (Prime et al., 2023), are integral to fostering higher IQ in children. The importance of maternal education and socioeconomic status further elucidates the indirect pathways through which parental characteristics influence cognitive development, highlighting the mediating role of maternal speech, expectations, and the quality of home interaction (Davis-Kean, 2005). Children often spend more time with their mothers, suggesting that environmental factors linked to maternal IQ and education might exert a stronger effect (Li and Guo, 2023). Apart from parental participants, the extent of a father’s involvement is related to socioeconomic class, with fathers from higher socioeconomic classes spending more time with their children (Rollè et al., 2019). Existing studies also indicate that aspects of maternal involvement, such as a mother’s education level and her own IQ, have a positive influence on the cognitive development and IQ of children (Meador et al., 2011; Lean et al., 2018). There remains a lack of definitive evidence elucidating the direct correlation between the amount of time mothers or fathers spend with their children and its impact on the children’s cognitive function. What is important in a child’s cognitive development is good quality parent–child relationships, with educational aspects, moreover a long period of time, and might not be explained simply by parental gender (mother or father) or parenting time alone (Li and Guo, 2023).

The study’s focus was solely on the quantity of time parents spent with their children, neglecting other crucial aspects like the quality of interactions or trust-building activities. The relationship with parents is not directly comparable to social capital, which examines networks of trust with others, and parental time, as it does not measure qualities such as trust. This focus might overlook significant elements of parental involvement that could impact child development and future well-being. Consequently, further investigations should consider the impact of the quality of parent–child interactions on child well-being and intelligence. Besides, the cross-sectional design of this study could not show the direct causal relationships between childhood social capital and adult subjective well-being. Although our investigation considers the influence of past experiences on present life conditions, it primarily relies on participants’ retrospective accounts, which inherently carries a risk of recall bias. This methodological approach restricts our capacity to fully delineate the longitudinal impact of social capital. Cohort studies, which follow individuals over extended periods, would provide a more robust framework for examining these temporal dynamics and their causal implications. Additionally, socioeconomic status (SES), which includes parental education levels might be a potential confounding variable because SES including parental education level and environmental factors such as neighborhood quality is associated with better developmental outcomes for children (Leventhal and Brooks-Gunn, 2000; Conger and Donnellan, 2007). Future research should incorporate detailed SES and broader environmental influence measures to comprehensively analyze its impact on social capital. Moreover, the study’s focus on university students in Japan presents limitations regarding cultural and socioeconomic diversity. Since university students often represent certain socioeconomic segments, this demographic might not fully capture the broader population’s diversity. The relative homogeneity in cultural and economic backgrounds among this group could influence the dynamics between social capital (SC) and subjective well-being (SWB). Therefore, it is important to consider that these findings might be more reflective of this specific group, highlighting the need for further research across a wider range of demographics. Finally, this research solely focused on SWB, omitting objective well-being metrics, which may not capture the full spectrum of well-being. Objective well-being, incorporating factors like financial stability and physical health, complements subjective reports to provide a more comprehensive picture of the relationship between social capital and well-being. These limitations indicate that the interpretations of our findings should be approached with caution. Future studies should strive to overcome these constraints by integrating longitudinal designs, a broader range of samples, and a blend of both subjective and objective well-being measures.

Our study provides valuable insights into the effects of childhood social connections, parental time, and social capital on adult well-being and intelligence quotient among Japanese university students. These findings have implications for understanding the long-term effects of early social experiences on individuals’ well-being and cognitive development.

## Data availability statement

The data that supports the findings of this study is available upon reasonable request to the corresponding author.

## Ethics statement

The studies involving humans were approved by Research Ethics Board of Tohoku University. The studies were conducted in accordance with the local legislation and institutional requirements. The participants provided their written informed consent to participate in this study.

## Author contributions

CH: Conceptualization, Data curation, Formal analysis, Funding acquisition, Investigation, Methodology, Project administration, Resources, Software, Supervision, Validation, Visualization, Writing – original draft, Writing – review & editing. ZY: Writing – original draft, Writing – review & editing. RT: Writing – review & editing. JW: Writing – review & editing. KM: Writing – review & editing. KH: Formal analysis, Investigation, Software, Writing – review & editing. TM: Data curation, Formal analysis, Validation, Visualization, Writing – original draft, Writing – review & editing.
